# Use of botulinum toxin in Frey's syndrome

**DOI:** 10.1002/ccr3.2019

**Published:** 2019-01-31

**Authors:** Francesco Freni, Francesco Gazia, Ferdinando Stagno d’Alcontres, Bruno Galletti, Francesco Galletti

**Affiliations:** ^1^ Department of Adult and Development Age Human Pathology “Gaetano Barresi”, unit of Otorhinolaryngology University of Messina Messina Italy

**Keywords:** botulinum toxin, Frey syndrome, Mikulicz syndrome, Minor test, parotidectomy, Sjogren syndrome

## Abstract

Frey's syndrome is the most common adverse event after parotidectomy, and usually, it appears after 6 months. In our case, symptoms appear 20 years from surgery, an uncommon condition. Intralesional botulinum toxin gives excellent results in therapy, regardless of the time elapsed between surgery and the first treatment.

## 1 INTRODUCTION

We present a case of a woman operated 20 years ago of bilateral parotidectomy that developed Frey's syndrome (FS). We try to explain the causes of the delay request for therapy in FS. We got the disappearance of symptoms even after 20 years from surgery thanks to botulinum toxin injection.

Frey's syndrome, also named auriculotemporal syndrome, is described as facial sweating and redness during meals following traumas or surgeries in the region of the parotid gland.^1^ Injury to the auriculotemporal nerve resulting from parotidectomy or parotid region trauma might damage parasympathetic and sympathetic fibers, and the parotid gland. The FS appears to be secondary to the abnormal reinnervation of the sweat glands and cutaneous vessels of the auricular‐temporal and large auricular nerve distribution territories by parasympathetic fibers, normally destined for the parotid and injured by the surgical intervention.^2^ Botulinum toxins, produced by Clostridium botulinum, are a family of neurotoxins that includes several subtypes. Botulinum toxin type A (BTXA) is the most common subtype, which causes blockage of neurotransmission by preventing the release of acetylcholine to nerve endings.^3^, ^4^

## 2 CASE REPORT

We present the case of a 42‐year‐old woman operated 20 years ago of bilateral parotidectomy for S. of Mikulicz. Her past medical history is pertinent for endoscopic sinus surgery for chronic rhinosinusitis,^5^ vocal cord surgery for a benign cyst,^6^, ^7^ and benign paroxysmal positional vertigo.^8^ She develops the signs of FS a few months before arriving in our unit, with reddening and sweating of the facial cutaway during the stimuli that produce salivation.

**Figure 1:**
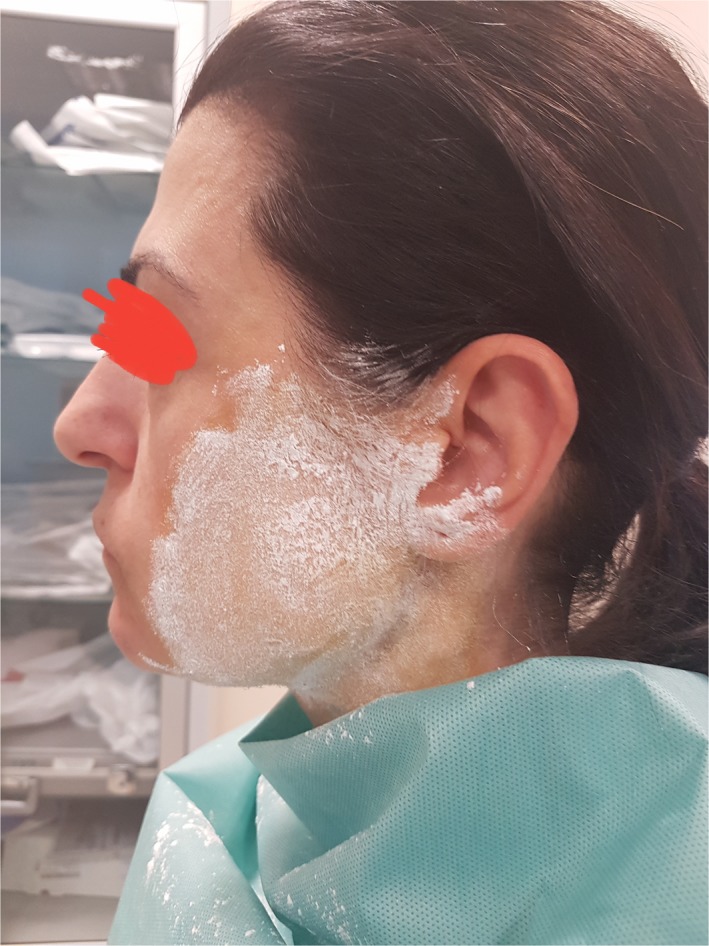
Minor test, the starch powder was applied on the dried iodine covered area

**Figure 2:**
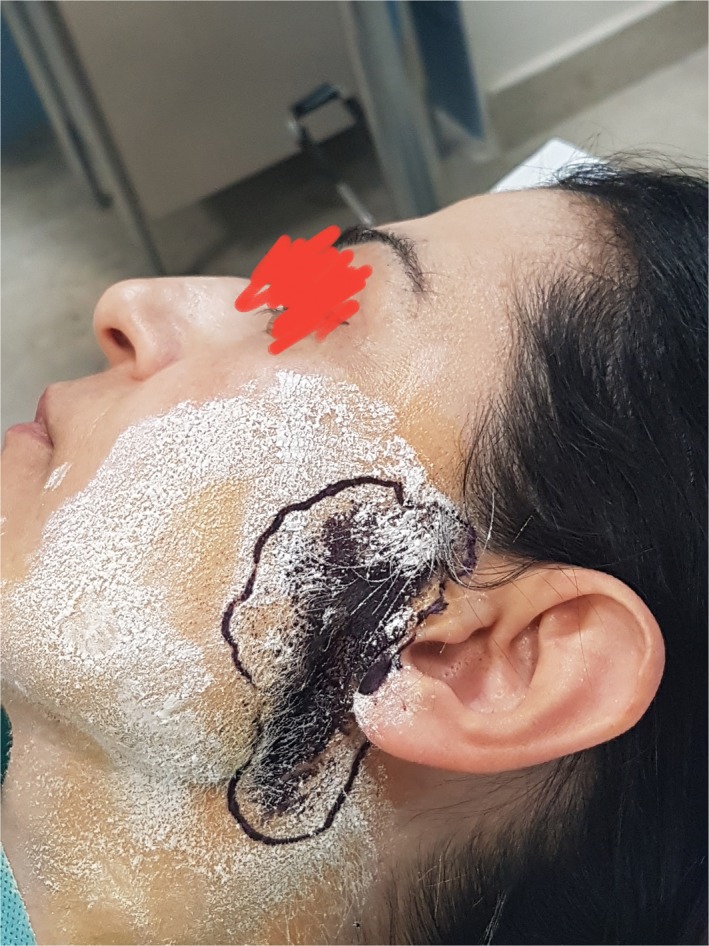
Minor test positive, brown‐violet areas appear on the skin

We diagnosed the FS with the Minor test. Preauricular area and cheek were covered with an iodine solution (15.0 g iodine, 100.0 g castor oil, 900.0 mL 70% alcohol). Then, the starch powder was applied on the dried iodine covered area (Figure 1). For the visualization of the symptoms, patients ate a candy for about 8 minutes. When brown‐violet areas appear on the skin, the test is considered positive^9^ (Figure 2).

**Figure 3:**
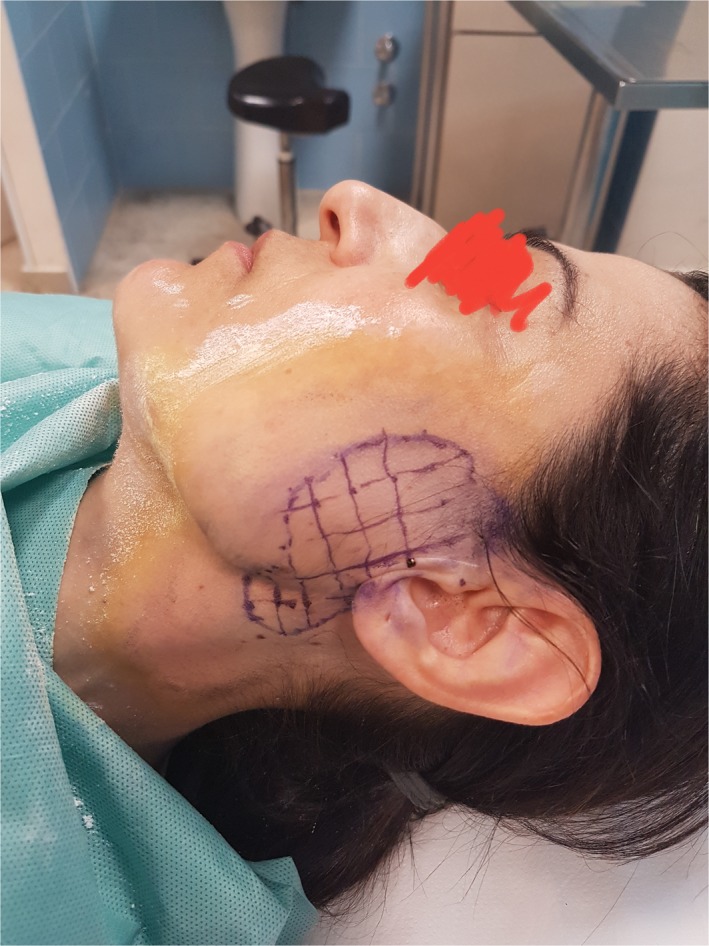
The area was subdivided into squares of 1 cm^2^ for a better distribution of the drug

The injection of the affected area of botulinum toxin type A Allergan 50 UI/mL is carried out. For treatment, the area was subdivided into squares of 1 cm^2^ for a better distribution of the drug (Figure 3). About 4.0 U of BTXA per cm^2^ were injected intracutaneously. The subjective absence of sweating and other symptoms of the syndrome defined the success of treatment. We performed a follow‐up at 1, 3 months, and then every 6 months to evaluate the possible disadvantages that may occur: dry mouth, weakening of the facial muscles, eyelid ptosis, facial paralysis, as well as short‐term local reactions of pain, edema, erythema, and ecchymosis. After about 25 months from the first injection, the patient will revert the signs of FS, which is confirmed by the repetition of the Minor test. We repeated the injection of botulinum. To date, after three years since the last botulinum injection, with a semi‐annual follow‐up, there are no signs of FS.

## 3 DISCUSSION

Frey's syndrome occurs more frequently after a parotid demolition surgery or after extensive trauma of the parotid region.^10^, ^11^, ^12^, ^13^, ^14^ The incidence after parotidectomy intervention is about 45%. For the treatment of FS, we can distinguish preventive surgical methods:

- Section of the efferent portion of the reflex arc (temporal auricular nerve, tympanic plexus, and intracranial portion of the glossopharyngeal nerve).
- Creations of sternocleidomastoid muscle flaps or transplant interposition of the fascia lata after parotidectomy.
- Creation of facial flaps of the superficial temporal artery.
- Creation of a subsurface aponeurotic flap.

The aim of surgical prevention is to create a barrier between the parasympathetic postganglia nerves of the parotid and the sweat glands of the overlying facial layer.^15^, ^16^, ^17^ We could not choose these therapeutic strategies because the patient was not operated in our unit.

There are also topical treatments to be used when the first symptoms appear for example, the topical application of anticholinergics and antiperspirants, and the intradermal injection of botulinum toxin.^18^

Creams based on atropine and scopolamine have shown poor and transient results, although there are no randomized trials that demonstrate these results.^19^

Nowadays, the intracutaneous injection of BTXA is the treatment of choice for FS because it appears simple, effective, reliable, fast, and without any apparent side effects.^9^ Alternative topical therapy with other substances such as aluminum chloride or epoetin, for the stimulation of angiogenesis and wound repair after traumas,^20^ is considered inferior to BTXA therapy due to difficulties in dose control and unclear long‐term side effects.^9^ In a recent review of Xie et al, the mean duration of effect varied from 3 to 20 months. The botox concentrations were between 16 and 75 U/mL, and the maximal dose used in per participants was 380 U without serious side effects. The distances between the two injection sites were 1, 1.5, or 2 cm. The botox concentration ranged from 2.0 to 5.0 U/cm^2^. Repeated botulinum toxin injections seem to promote a reduction in the severity of the symptoms and the extension of the treated area, as well as space out the period between recurrences, like in our case. One possible explanation would be the atrophy of the eccrine glands, inhibited for long periods.^21^ The mean time interval from surgery or trauma to the first injection was 4‐5 years. The longest time interval described in the literature is 33.8 years.^9^ In our case, the first symptoms of FS appear after 20 years from surgery. There are different causes that can explain the delay request for therapy in FS^22^:

- The improvement of other postoperative sequelae, such as facial paresis or loss of sensitivity, may detect permanent symptoms of FS.
- The possible slow enlargement of the sweating area after surgery may lead to a delayed subjective awareness of the syndrome accompanied by a delayed treatment request.
- The longer reinnervation time of the parasympathetic fibers may be another reason for delaying the onset of symptoms.
- Alterations in repair process by second intention after surgery, with formation of denser connective tissue that hinders reinnervation.

These causes slow down the appearance of Frey's syndrome, but the therapeutic action of botulinum is always guaranteed. In fact, BTXA only acts after synapses have formed between the parasympathetic parotid fibers and the sweat glands, blocking the release of acetylcholine.

Further studies are needed to find out the possible reasons for delayed therapy in patients with Frey syndrome. In our case, we got the disappearance of symptoms using BTXA even after 20 years from surgery. These theories confirm that the time elapsed between surgery and treatment with botulinum does not appear discriminating for the successful outcome of therapy.

## 4 CONCLUSION

There are different causes that can explain the delay request for therapy in FS. In our case, symptoms appear 20 years from surgery. Our report confirms that the time elapsed between surgery and treatment with botulinum does not appear discriminating for the successful outcome of therapy.

## CONFLICT OF INTEREST

None declared.

## AUTHORS CONTRIBUTION

FF: developed the concept and design of the study. FGazia: is corresponding author, was preparing the manuscript. FSdA: performed follow‐up examinations. BG: was treating the patient. FGalletti: critically revised the manuscript for important intellectual content and gave the final approval of the version to be submitted.

## References

[1] Linder TE , Huber A , Schmid S . Frey’s syndrome after parotidectomy: a retrospective and prospective analysis. Laryngoscope. 1997;107:1496‐1501. 9369396 10.1097/00005537-199711000-00013

[2] Ford FR , Woodhall R . Phenomena due to misdirection of regenerating fibres of cranial, spinal and automatic nerves, clinical observations. Arch Surg. 1938;36:480‐496.

[3] Tugnoli V , Marchese Ragona R , Eleopra R , et al. The role of gustatory flushing in Frey’s syndrome and its treatment with botulinum toxin type A. Clin Auton Res. 2002;12:174‐178. 12269549 10.1007/s10286-002-0026-x

[4] Xie S , Wang K , Xu T , Guo XS , Shan XF , Cai ZG . Efficacy and safety of botulinum toxin type A for treatment of Frey's syndrome: evidence from 22 published articles. Cancer Med. 2015;4(11):1639‐1650. 26310612 10.1002/cam4.504 PMC4673990

[5] Galletti B , Gazia F , Freni F , Sireci F , Galletti F . Endoscopic sinus surgery with and without computer assisted navigation: A retrospective study. Auris Nasus Larynx. 2018. 10.1016/j.anl.2018.11.004 30528105

[6] Galletti B , Freni F , Cammaroto G , Catalano N , Gangemi G , Galletti F . Vocal outcome after CO2 laser cordectomy performed on patients affected by early glottic carcinoma. J Voice. 2012;26(6):801‐805. 22717493 10.1016/j.jvoice.2012.01.003

[7] Marseglia L , D'Angelo G , Impellizzeri P , et al. Neonatal stridor and laryngeal cyst: Which comes first? Pediatr Int. 2017;59(1):115‐117. 28102625 10.1111/ped.13192

[8] Ciodaro F , Mannella VK , Nicita RA , et al. Therapeutic efficacy of the Galletti‐Contrino manoeuvre for benign paroxysmal positional vertigo of vertical semicircular canals in overweight subjects. Eur Arch Otorhinolaryngol. 2018;275(10):2449‐2455. 30097716 10.1007/s00405-018-5086-1

[9] Jansen S , Jerowski M , Ludwig L , Fischer‐Krall E , Beutner D , Grosheva M . Botulinum toxin therapy in Frey's syndrome: a retrospective study of 440 treatments in 100 patients. Clin Otolaryngol. 2017;42(2):295‐300. 27513469 10.1111/coa.12719

[10] Galletti B , Bruno R , Catalano N , Cammaroto G , Freni F . Follicular carcinoma on a radio‐treated ectopic lingual thyroid. Chirurgia (Turin). 2016;29(3):88‐91.

[11] Freni F , Galletti B , Galletti F , Dionigi G . Improved outcomes for papillary thyroid microcarcinoma care: active surveillance and case volume. Ther Adv Endocrinol Metab. 2018;9(7):185‐186. 29977496 10.1177/2042018818773609 PMC6022973

[12] Galletti B , Mannella VK , Santoro R , et al. Ear, nose and throat (ENT) involvement in zoonotic diseases: a systematic review. J Infect Dev Ctries. 2014;8(1):17‐23. 24423708 10.3855/jidc.4206

[13] Galletti B , Mannella VK , Santoro R , et al. Malignant external otitis. A case series from an Italian Tertiary‐Care Hospital. Acta Medica Mediterranea. 2014;30(6):1317‐1323.

[14] Trovato M , Ruggeri RM , Guzzo E , et al. Expression of p53 and isoforms in beningn and malignant lesions of the head and neck. Histol Histopathol. 2017;32(4):371‐377. 27411919 10.14670/HH-11-802

[15] Zhang M , Cao SW , Liu JM . The study of prevention the gustatory sweating syndrome and facial contour deformity with sternocleidomastoid muscle flaps in the parotidectomy. Lin Chung Er Bi Yan Hou Tou Jing Wai Ke Za Zhi. 2016;30(6):482‐484. 29871045 10.13201/j.issn.1001-1781.2016.06.016

[16] Rubinstein RY , Rosen A , Leeman D . Frey syndrome: treatment with temporoparietal fascia flap interposition. Arch Otolaryngol Head Neck Surg. 1999;125(7):808‐811. 10406323 10.1001/archotol.125.7.808

[17] Ahmed OA , Kolhe PS . Prevention of Frey's syndrome and volume deficit after parotidectomy using the superficial temporal artery fascial flap. Br J Plast Surg. 1999;52(4):256‐260. 10624290 10.1054/bjps.1998.0137

[18] Clayman MA , Clayman SM , Seagle MB . Review of the surgical and medical treatment of Frey syndrome. Ann Plast Surg. 2006;57:581‐584. 17060744 10.1097/01.sap.0000237085.59782.65

[19] Li C , Wu F , Zhang Q , Gao Q , Shi Z , Li L . Interventions for the treatment of Frey's syndrome. Cochrane Database Syst Rev. 2015;(3):CD009959. 25781421 10.1002/14651858.CD009959.pub2 PMC10799668

[20] Irrera N , Bitto A , Pizzino G , et al. Epoetin alpha and epoetin zeta: a comparative study on stimulation of angiogenesis and wound repair in an experimental model of burn injury. BioMed Res Int. 2015;2015:968927. 26146639 10.1155/2015/968927 PMC4471383

[21] Martos Díaz P , Bances del Castillo R , Mancha de la Plata M , et al. Clinical results in the management of Frey's syndrome with injections of Botulinum toxin. Med Oral Patol Oral Cir Bucal. 2008;13:E248‐E252. 18379450

[22] Blitzer A , Sulica L . Botulinum toxin: basic science and clinical uses in otolaryngology. Laryngoscope. 2001;111(2):218‐226. 11210864 10.1097/00005537-200102000-00006

